# The novel C5 protein from tomato yellow leaf curl virus is a virulence factor and suppressor of gene silencing

**DOI:** 10.1007/s44154-022-00044-3

**Published:** 2022-04-02

**Authors:** Siwen Zhao, Pan Gong, Yanxiang Ren, Hui Liu, Hao Li, Fangfang Li, Xueping Zhou

**Affiliations:** 1 State Key Laboratory for Biology of Plant Diseases and Insect Pests, Institute of Plant Protection, Chinese Academy of Agricultural Sciences, Beijing, 100193 China; 2 State Key Laboratory of Rice Biology, Institute of Biotechnology, Zhejiang University, Zhejiang, 310058 Hangzhou China

**Keywords:** Tomato yellow leaf curl virus, C5, Hypersensitive response, Post-transcriptional gene silencing, Transcriptional gene silencing, Pathogenicity determinant

## Abstract

Tomato yellow leaf curl virus (TYLCV) is known to encode 6 canonical viral proteins. Our recent study revealed that TYLCV also encodes some additional small proteins with potential virulence functions. The fifth ORF of TYLCV in the complementary sense, which we name C5, is evolutionarily conserved, but little is known about its expression and function during viral infection. Here, we confirmed the expression of the TYLCV C5 by analyzing the promoter activity of its upstream sequences and by detecting the C5 protein in infected cells by using a specific custom-made antibody. Ectopic expression of *C5* using a potato virus X (PVX) vector resulted in severe mosaic symptoms and higher virus accumulation levels followed by a burst of reactive oxygen species (ROS) in *Nicotiana benthamiana* plants. C5 was able to effectively suppress local and systemic post-transcriptional gene silencing (PTGS) induced by single-stranded GFP but not double-stranded GFP, and reversed the transcriptional gene silencing (TGS) of GFP. Furthermore, the mutation of C5 in TYLCV inhibited viral replication and the development of disease symptoms in infected plants. Transgenic overexpression of C5 could complement the virulence of a TYLCV infectious clone encoding a dysfunctional C5. Collectively, this study reveals that TYLCV C5 is a pathogenicity determinant and RNA silencing suppressor, hence expanding our knowledge of the functional repertoire of the TYLCV proteome.

**Supplementary Information:**

The online version contains supplementary material available at 10.1007/s44154-022-00044-3.

## Introduction

Geminiviruses (family *Geminiviridae*) are plant single-stranded circular DNA (ssDNA) viruses, which infect economically important crops including tomato, corn, and cotton, causing worldwide agricultural losses (Moffat et al., 1999; Glick et al., 2008; Navas-Castillo et al., 2011). Geminiviruses can be divided into fourteen genera based on host range, insect vectors, and genomic organization (Walker et al., 2021). Among these genera, *Begomovirus* is the largest genera in the family *Geminiviridae* and contains more than 400 species that are transmitted by whiteflies (Brown et al., 2015). Begomoviruses can have either monopartite (a single circular ssDNA molecule) or bipartite (two circular ssDNA molecules) genomes (Rojas et al., 2005). Many monopartite begomoviruses are associated with betasatellites in the field, which are essential for the induction of typical disease symptoms (Zhou, 2013; Li et al., 2018). For example, the βC1 and βV1 proteins, encoded by tomato yellow leaf curl China betasatellite (TYLCCNB), are important pathogenicity factors during the complex infection of tomato yellow leaf curl China virus (TYLCCNV) and TYLCCNB (Li et al., 2018; Hu et al., 2020; Gui et al., 2022).

It is widely accepted that monopartite begomoviruses encode 6 canonical viral proteins. The coat protein (CP) encoded by the V1 ORF from the viral sense-strand DNA is necessary for the packaging of the viral DNA and the spread by insect vectors (Harrison et al., 2002). The V2 ORF encodes the V2 protein, which plays a role in the movement of the virus (Zhao et al., 2020). V2 is also an RNA silencing suppressor of post-transcriptional gene silencing (PTGS) and transcriptional gene silencing (TGS). The V2 protein of tomato yellow leaf curl virus (TYLCV) suppresses PTGS and TGS through its physical interaction with SGS3 and AGO4, respectively (Glick et al., 2008; Wang et al., 2020). The replication associated protein (Rep) encoded by the C1 ORF from the complementary strand is essential for reprograming the cell cycle and mediating rolling circle replication (RCR) of the viral genome (Hanley-Bowdoin et al., 2013; Basak, 2016). The transcription activator protein (TrAP) is encoded by the C2 ORF, and can activate the transcription of late genes on the viral genome (Sunter & Bisaro, 1991). C2 can also suppress PTGS, TGS, protein ubiquitination, and jasmonic acid (JA) signaling (Lozano-Duran et al., 2011; Luna et al., 2012; Rosas-Diaz et al., 2016). The replication enhancing protein (REn) is encoded by the C3 ORF, and is important for viral replication by selectively recruiting DNA polymerase δ over ε to favour a productive replication (Wu et al., 2021). The C4 protein has multiple roles in development of disease symptoms, participation in viral movement, and suppression of host DNA methylation and RNA silencing (Rojas et al., 2001; Kon et al., 2007; Luna et al., 2012; Mei et al., 2020a; Mei et al., 2020b; Medina-Puche et al., 2021). Therefore, geminiviruses have evolved diverse mechanisms to deploy viral proteins to create a favorable environment for viral replication and infection.

A recent report has indicated that geminiviral genomes contain additional ORFs besides the canonical ones described to date. For example, the novel V3 protein of TYLCV, is a Golgi- and partially endoplasmic reticulum (ER)-localized protein, which functions as an RNA silencing suppressor and a viral cellular movement protein (Gong et al., 2021; Gong et al., 2022). These previously neglected ORFs frequently encode proteins that are phylogenetically conserved (Gong et al., 2021). Consistent with this finding, the functionality of the AC5/C5 protein encoded by the fifth ORF from the complementary strand in the DNA-A component of several monopartite and bipartite begomoviruses has also been previously proven (Li et al., 2015; Li et al., 2021). The AC5/C5 ORF encodes a protein of about 100 amino acids, is located downstream of the C3 ORF, and overlaps with a portion of the V1 ORF on the viral sense-strand. The known AC5/C5 proteins are reported to be multifunctional. For example, the bipartite begomovirus mungbean yellow mosaic India virus (MYMIV) AC5 plays a role in geminiviral DNA replication in a yeast model system (Raghavan et al., 2004). Further work has shown that MYMIV AC5 is a pathogenicity determinant and functions as a suppressor of TGS and PTGS (Li et al., 2015). The AC5 ORF of tomato chlorotic mottle virus (ToCMoV) and watermelon chlorotic stunt virus (WmCSV) encodes proteins of 250 and 255 amino acids, respectively, which are not essential for the virus infection cycle, according to mutant analysis (Kheyr-Pour et al., 2000; Fontenelle et al., 2007), indicating that the role of AC5/C5 might be diverse in different geminiviruses.

TYLCV is a monopartite begomovirus and is responsible for serious yield losses in tomato cultivation worldwide. Using cap-snatching of rice stripe virus (RSV) in TYLCV-infected *Nicotiana benthamiana* plants, 21 transcriptional initiation sites located in the TYLCV genome were identified (Lin et al., 2017), indicating that TYLCV might encode multiple ORFs that have so far been neglected. Our recent work confirmed that TYLCV contains additional small ORFs, encoding proteins with different subcellular localization patterns (Gong et al., 2021). Among these small ORFs, ORF1, which we name C5, is evolutionarily conserved in the family *Geminiviridae*, and is located in the nucleus and cytoplasm (Gong et al., 2021). However, its expression and function during viral infection is not investigated. Here we confirmed the expression of C5 in the context of TYLCV infection, and found that this protein is involved in the development of viral symptoms and viral DNA accumulation through mutation and complementation assays. We also revealed that C5 functions as an RNA silencing suppressor with the ability to suppress both PTGS and TGS. These findings uncover a novel TYLCV-encoded protein that contributes to the virus infection, and expands our knowledge of the proteins encoded by small ORFs in TYLCV.

## Results

### Sequence analysis of C5 and detection of the C5 transcript and protein

**Fig. 1:**
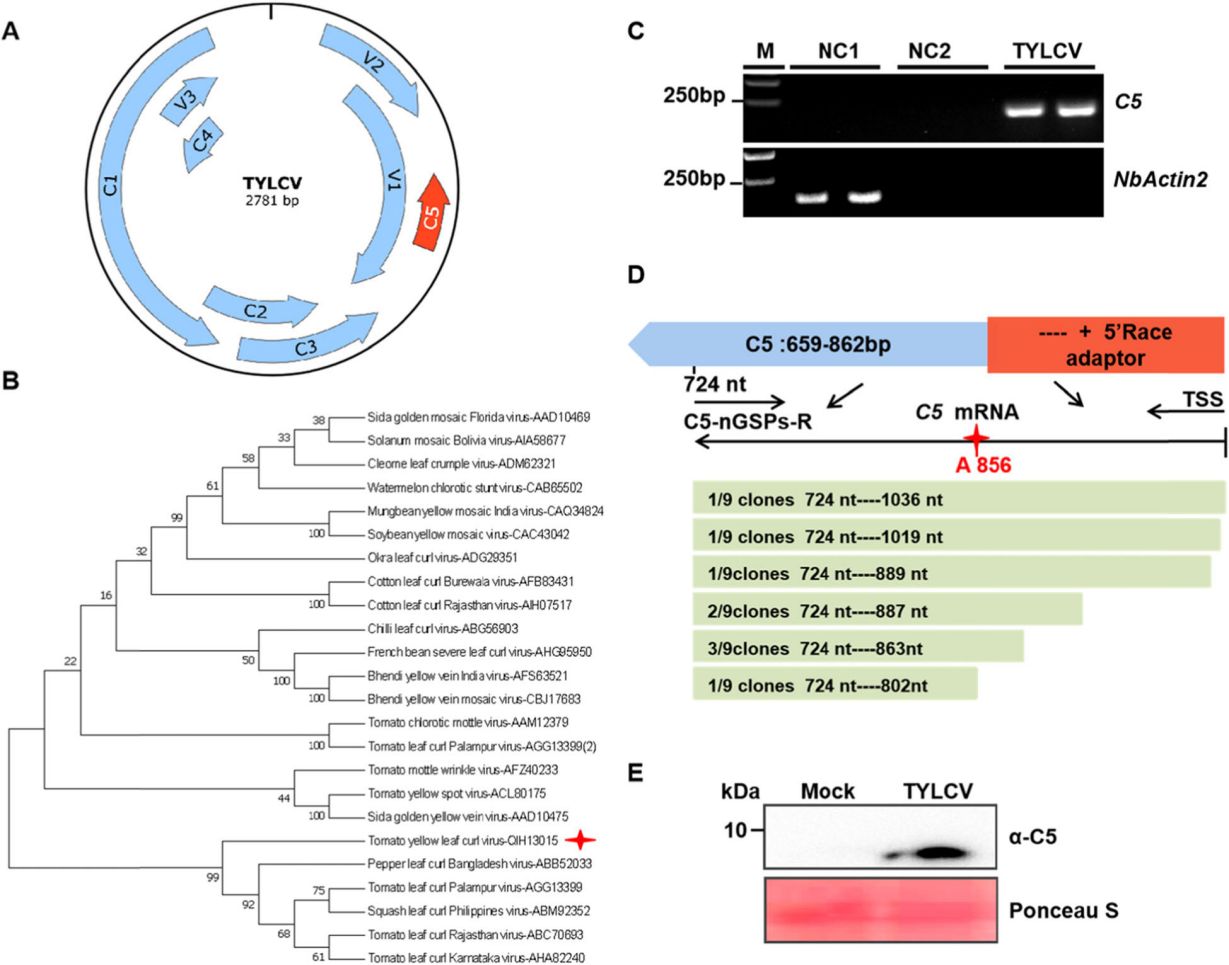
Sequence analyses of the TYLCV C5 protein. **A** Schematic representation of the TYLCV genome. **B** Phylogenetic analysis of C5 amino acid (aa) sequences of representative begomoviruses. The amino acid sequences of C5 proteins from 23 begomoviruses viruses were aligned using by ClustalW method with MEGA7. **C** RT-PCR analysis of C5 transcripts from TYLCV-infected or mock-inoculated *N. benthamiana* plants. M: DNA ladder marker. NC1: negative control 1 (reverse-transcription of total RNA extracted from the mock-inoculated plants with RT Primer mix of random 6 mers and oligo dT primer. NC2: negative control 2 (reverse-transcription of total RNA extracted from the mock-inoculated plants with *C5*-specific primers). TYLCV: reverse-transcription of total RNA extracted from the TYLCV-infected plants with *C5*-specific primers. *C5*: PCR products of *C5* with a pair of *C5*-specific primers. *NbActin2*: PCR products of *NbActin2* with a pair of *NbActin2*-specific primers. This experiment was repeated three times with similar results; representative images are shown. **D** Transcriptional start site analysis of TYLCV C5 by 5′ RACE. TSS: transcription start site. A856: C5 TSS was captured by RSV cap-snatching (Lin et al., 2017). **E** Western blot analysis of total protein from the TYLCV-infected *N. benthamiana* leaves at 72 hpi with anti-C5 antibodies, Mock: *N. benthamiana* plants infiltrated with an *A. tumefaciens* clone containing the pCambia 2300 vector. Three plants were analyzed for each treatment. This experiment was repeated three times with similar results. Ponceau S staining of the large RuBisCO subunit serves as loading control

Our recent study shows that TYLCV encodes additional small ORFs (Gong et al., 2021). The TYLCV C5 ORF (nucleotide coordinates 862--659) encodes a polypeptide of 67 amino acids (aa), which overlaps with the partial V1 ORF, but it is transcribed in the opposite direction (Fig. 1A). To further investigate the evolutionary relationship of C5 sequences from different begomoviruses, we aligned the C5 aa sequences from 23 different geminiviruses using the neighbor-joining method with MEGA7 and generated a phylogenetic tree. The result indicates that the TYLCV C5 is most closely related to that of pepper leaf curl Bangladesh virus (PepLCBV) (Fig. 1B). Of note, the C5 ORF is also conserved in different begomoviruses (47/58) from the ‘New World’ and ‘Old World’ (Fig. S1A). To determine whether the C5 ORF is transcribed during viral infection, total RNA from the TYLCV-infected or mock-inoculated leaves of *N. benthamiana* plants were extracted for RT-PCR assays using C5-specific primers. As shown in Fig. 1C, the C5 RNA transcripts were only detected in TYLCV-infected plants of *N. benthamiana*, but not in mock-inoculated plants. Using the absolute quantitative analysis by specific primers listed in the Supplemental Table 1, we found that the presence of TYLCV C5 transcripts in infected leaves and other known viral transcripts (V1, C1 and C4) were also detected as positive controls (Fig. S1B). The C5 transcript was further identified by the 5′ rapid amplification of cDNA ends (RACE), which showed that the transcriptional initiation site was located between 60 and 174 nt upstream of the translation initiation codon of C5 ORF (Fig. 1D). Three of nine sites (site 863) identified by RACE are close to A856 (Fig. 1D), which was previously identified using cap-snatching of RSV (Lin et al., 2017). In addition, C5 was also expressed in *Escherichia coli* cells as a 6X Histidine fusion. The C5-His fusion protein was purified using a nickel affinity column (Merck, Darmstadt, Germany) and eluted with buffer containing 200 mM imidazole according to the manufacturer’s manual (Fig. S1C). The purified protein was then used to immunize rabbits for generating polyclonal antibodies, and the purified C5-His fusion protein was successfully detected by anti-C5 antibodies (Fig. S1C). Using the purified anti-C5 antibodies, we could also detect the presence of the C5 protein in TYLCV-inoculated leaves of *N. benthamiana* at 72 h post inoculation (hpi) (Fig. 1E). Taken together, these results confirm that the C5 ORF is expressed in planta from its genomic context.

### Promoter activity analysis of the upstream sequences of C5

**Fig. 2:**
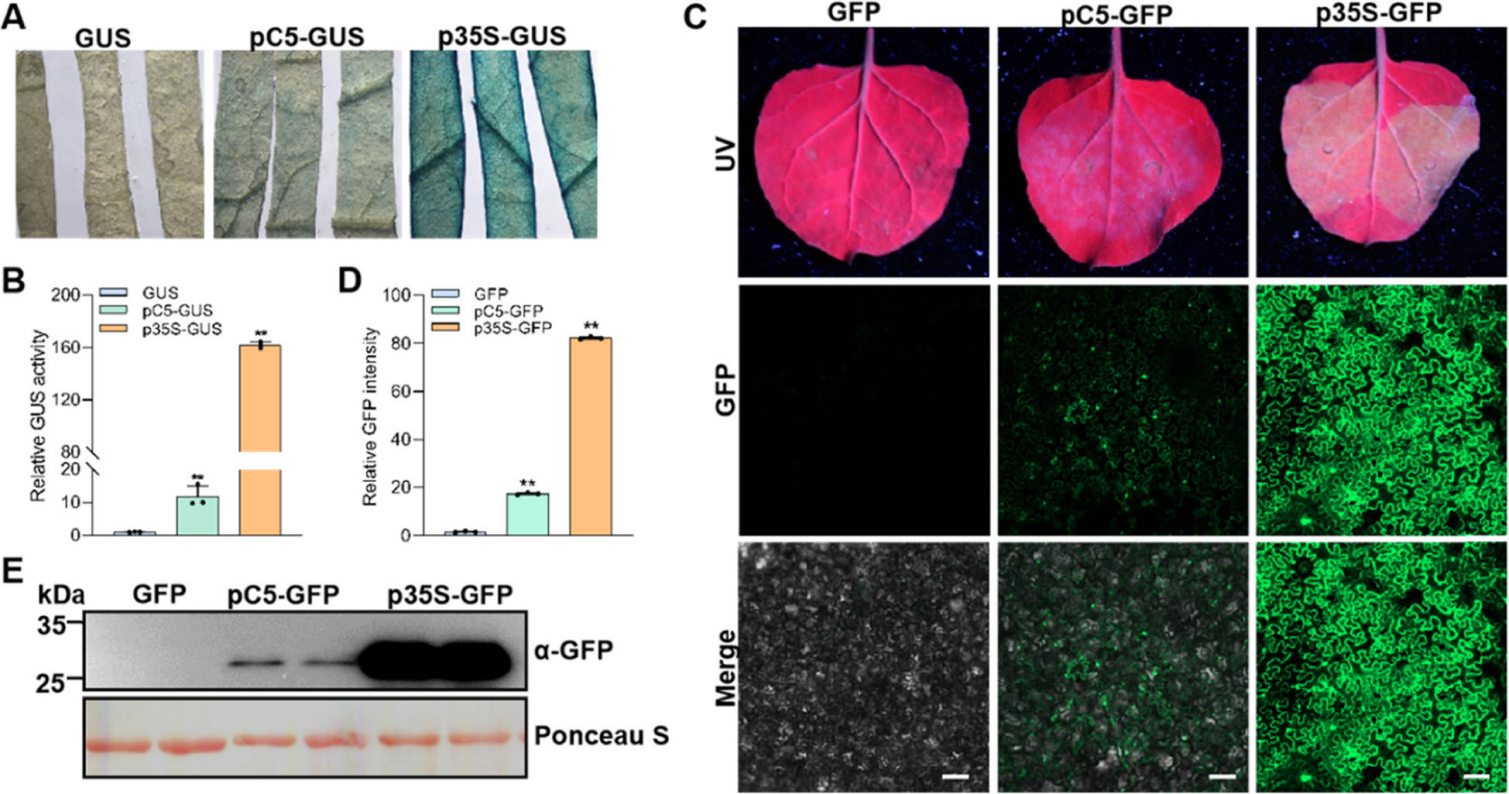
Expression of GUS and GFP can be driven by the C5 promoter sequence. **A** The activity of the promoter sequence of C5 analyzed by GUS staining; the 35S promoter is used as positive control. **B** Quantification of relative GUS activity in samples from (**A**). Data are the mean of three independent biological replicates. Error bars represent SD (*n* = 3). **C** The C5 and 35S promoters drive the expression of GFP. The GFP fluorescence was observed in transiently transformed *N. benthamiana* leaves at 2 dpi using confocal microscope. Bar: 100 μm. **D** Quantification of relative GFP intensity in samples from (**C**). The intensity of GFP fluorescence was measured with the ZEN3.1 software with the method of mean intensity in the same area. **E** Western blot analysis of total protein extracted from (**C**) with anti-GFP antibodies. Ponceau S staining of the large RuBisCO subunit serves as loading control. This experiment was repeated three times with similar results; representative results were shown

A prerequisite for the C5 ORF to have a biological function is its expression in the context of viral infection. To further confirm expression of this gene, we cloned the ~ 500 nt TYLCV genomic sequences upstream of the C5 ORF and then fused it with GUS and GFP to generate two recombinant vectors: pC5-GUS and pC5-GFP. The pC5-GUS, pC5-GFP vectors, along with promoter-less negative controls (GUS, GFP) and positive controls containing the 35S promoter of cauliflower mosaic virus (CaMV) (p35S-GUS, p35S-GFP) were transformed into *Agrobacterium tumefaciens* and then agroinfiltrated into leaves of *N. benthamiana.* At 2 days post inoculation (dpi), the promoter activity was measured by comparing the activity of GUS and the intensity of the GFP fluorescence using GUS staining and confocal analyses, respectively. We found that this viral sequence could effectively activate the expression of GFP and GUS, leading to detectable GUS activity and observational GFP fluorescence (Fig. 2A-E). However, compared to the 35S promoter, the C5 promoter sequence displayed weaker activity in driving the expression of GFP or GUS (Fig. 2A-E). These results indicate that the 500 nt sequence upstream of the C5 ORF has promoter activity.

### C5 is a virulence determinant and elicits a burst of reactive oxygen species (ROS) in *N. benthamiana*

**Fig. 3:**
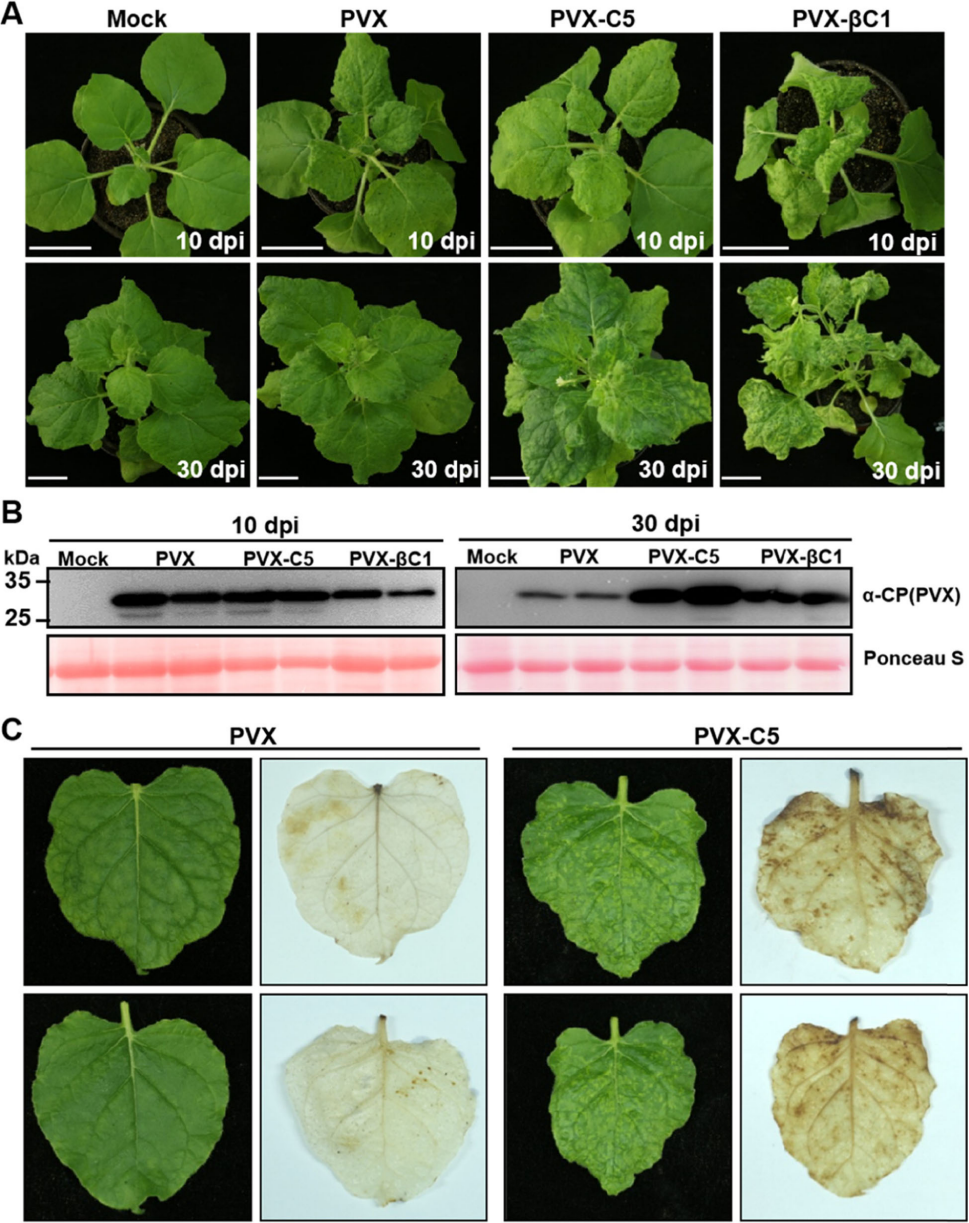
Symptoms exhibited by *N. benthamiana* plants following inoculation with PVX-C5. **A** Disease symptoms elicited on *N. benthamiana* plants by inoculation of Mock (infiltration buffer), PVX, PVX-C5, or PVX-βC1 (positive control) at 10 dpi and 30 dpi. Bar = 4 cm. **B** Western blot analysis of PVX CP accumulation with specific anti-PVX-CP antibodies in systemic leaves from (**A**). This experiment was repeated three times with similar results. Ponceau S staining of the large RuBisCO subunit serves as loading control. **C** Disease symptoms and DAB staining of the upper newly leaves of *N. benthamiana* plants infected by PVX and PVX-C5 at 15 dpi

Plus-strand RNA viruses are often used as expression vectors in plants, because they tolerate extra transgene insertion and expression without disrupting normal virus functions (Dickmeis et al., 2014). For example, a potato virus X (PVX)-based vector is suitable for the systemic expression of any gene of interest in PVX host plants. With the aim of assessing the biological relevance of the C5 protein in virus infection, we constructed a PVX-based recombinant vector carrying the *C5* gene (PVX-C5) for ectopic overexpression of this viral sequence. PVX-C5 was inoculated to 4-week-old *N. benthamiana* plants by agroinfiltration. *A. tumefaciens* cultures carrying the empty binary vector (Mock) or PVX were inoculated as negative controls, and the recombinant PVX vector expressing *βC1* gene of TYLCCNB (PVX-βC1) were inoculated as positive controls. The inoculated plants were maintained to monitor viral symptom appearance and analyze viral protein accumulation in upper new leaves. At 10 dpi, the symptoms of PVX-C5-inoculated plants were similar to those of PVX-inoculated control plants, which displayed obvious mosaic symptoms (Fig. 3A). However, PVX-C5 infected plants developed more severe mosaic, chlorosis, leaf deformity, and necrotic symptoms at 15 dpi. All 30 tested plants agroinoculated with PVX-C5 showed the aforementioned symptoms in systemically infected leaves at 30 dpi (Fig. 3A). However, PVX-infected plants showed symptom recovery at this time ((Fig. 3A). The positive control, PVX-βC1-infected plants, exhibited the typical βC1-associated leaf curling phenotype, in addition to the PVX mosaic symptom at 10 dpi and 30 dpi (Fig. 3A). To test the effect of C5 on the accumulation of PVX in infected plants, we analyzed the accumulation of the PVX coat protein (CP) by using anti-PVX-CP antibodies by western blot at 10 dpi and 30 dpi. The result shows that more PVX CP protein accumulated in PVX-C5 infected plants compared with PVX infected plants at 30 dpi, indicating that TYLCV C5 is a virulence factor that enhances PVX pathogenicity in *N. benthamiana* plants (Fig. 3B).

Meanwhile, we observed that the plants infected by PVX-C5, but not PVX, displayed obvious necrosis in newly emerged leaves after 15 dpi (Fig. 3C). Previous studies have shown that a burst of ROS in plants can lead to local production of programmed cell death (PCD) in leaves, thereby restraining further infection, which is a ubiquitous defense response to pathogens (Muhlenbock et al., 2008; Ruan et al., 2019). 3,3′-diaminobenzidine (DAB) staining is widely used to detect ROS production (e.g. H_2_O_2_ accumulation). Hence, we performed a DAB staining on systemic leaves of PVX- and PVX-C5-infected plants at 15 dpi. As shown in Fig. 3C, obvious brown precipitates appeared in the PVX-C5- but not in the PVX-infected leaves, suggesting that high levels of H_2_O_2_ accumulate in the former.

### C5 blocks local and systemic RNA silencing triggered by single-stranded GFP (ssGFP), but not by double-stranded GFP (dsGFP)

**Fig. 4:**
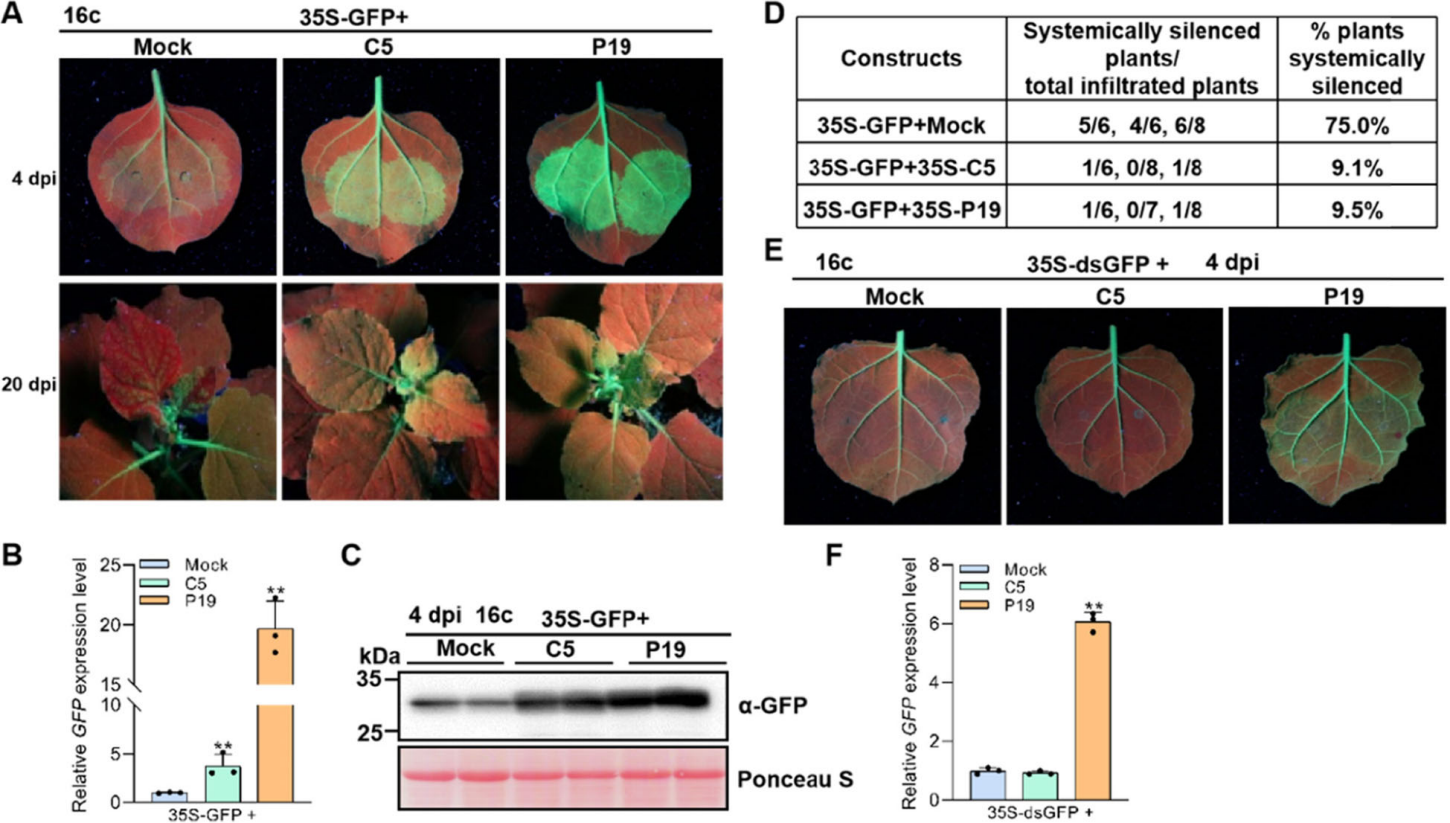
C5 suppresses single-stranded GFP-induced RNA silencing. **A** 16c *N. benthamiana* plants co-infiltrated with *A. tumefaciens* cultures expressing GFP (35S-GFP) and vector control (Mock), C5, or TBSV P19 (positive control), were photographed under UV light at 4 dpi and 20 dpi. **B** RT-qPCR analysis of relative *GFP* expression levels in the agroinfiltrated leaf patches from (**A**). Three plants were analyzed for each treatment; this experiment was repeated three times with similar results. Error bars represent ± SD (*n* = 3), and *NbActin2* was used as internal reference. **C** Western blot analysis of GFP protein accumulation in the agroinfiltrated leaf patches as indicated in (**A**). This experiment was repeated three times with similar results. Ponceau S staining of the large RuBisCO subunit serves as loading control. **D** The percentage of *N. benthamiana* 16c plants infiltrated with Mock, C5 or TBSV P19 showing systemic RNA silencing. At least 6 plants were used in each independent systemic silencing experiment; this experiment was repeated three times with similar results. **E** 16c *N. benthamiana* plants co-infiltrated with double-stranded GFP (35S-dsGFP) and Mock, C5 or P19, were photographed under UV light at 4 dpi. **F** RT-qPCR analysis of relative *GFP* expression levels in the agroinfiltrated leaf patches from (**E**). Three plants were analyzed for each treatment and this experiment was repeated three times with similar results. Error bars represent ± SD (*n* = 3) and *NbActin2* was used as internal reference. Student’s *t* test was used to statistically analyze each group of data, and double asterisks indicate significant statistical differences (**p < 0.01) between two treatments (**C** and **F**)

To establish a successful infection, almost all viruses have evolved mechanisms to counterattack RNA silencing by encoding at least one viral suppressor of RNA silencing (VSR) (Kon et al., 2007; Li et al., 2014; Li & Wang, 2019). To identify whether the C5 protein of TYLCV is a VSR, we agroinfiltrated the GFP-transgenic *N. benthamiana* 16c plants with mixed *A. tumefaciens* cultures carrying 35S:GFP with Mock, C5, or P19 in a ratio of 1:1. 35S-GFP could express ssGFP as an RNA silencing inducer, Mock is an empty vector used as a negative control, and P19 is a well-studied VSR of tomato bushy stunt virus, used as a positive control. At 4 dpi, we observed that GFP fluorescence intensity decreased substantially in the 35S-GFP with Mock co-infiltrated leaf patches, while it was enhanced in the 35S-GFP with C5 or with P19 co-infiltrated leaf patches under UV light (Fig. 4A and S2A). RT-qPCR and western blot analyses showed that the co-expression of P19 or C5 led to the more GFP RNA and higher protein accumulation, although the effect of C5 appeared to be weaker than that of P19 (Fig. 4B-C). The agroinfiltrated plants were maintained to examine systemic silencing in upper young leaves at 20 dpi. In the negative controls, the upper young leaves of ~ 75% infiltrated plants turned red under UV light, indicating systemic RNA silencing (Fig. 4A and D); however, the newly emerged leaves in more than 90% plants co-infiltrated with C5 and P19 remained green under UV light (Fig. 4A and D), suggesting that both C5 and P19 can suppress ssGFP-induced systemic RNA silencing.

To further confirm the ability of C5 to suppress RNA silencing, the viral vector PVX was also used to express C5. *A. tumefaciens* cultures harboring 35S-GFP and a negative control (PVX), a positive control (PVX-βC1), or PVX-C5, respectively, were co-infiltrated into 16c leaves. Consistent with our previous results, the expression of C5 from a PVX vector could also efficiently suppress ssGFP-induced local and systemic RNA silencing at 7 dpi and 20 dpi, respectively (Fig. S3A-C).

DsGFP-induced RNA silencing is also commonly used to analyze the suppression activity of VSRs (Li & Wang, 2019). To further clarify whether C5 interferes with the silencing triggered by dsRNA, we mixed the *A. tumefaciens* cultures harboring 35S-dsGFP (which expresses a dsRNA fragment of the *GFP* gene) with Mock, C5, or P19 at a ratio of 1:1 and infiltrated 16c *N. benthamiana* leaves. At 4 dpi, the infiltrated patches with Mock or C5 turned red due to the onset of RNA silencing, while the expression of P19 significantly enhanced GFP fluorescence in the infiltrated leaves (Fig. 4E and S2B). RT-qPCR analysis showed that the accumulation of GFP RNA in C5-expressing leaf patches was similar to that in Mock infiltrated leaf patches, which was significantly lower than that in P19-expressing leaf patches (Fig. 4F). This result suggests that C5 is unable to suppress the RNA silencing triggered by dsRNA. Taken together, these findings provide persuasive evidence that C5 is capable of suppressing ssGFP rather than dsGFP-induced RNA silencing in *N. benthamiana* plants.

### C5 acts as a TGS suppressor

**Fig. 5:**
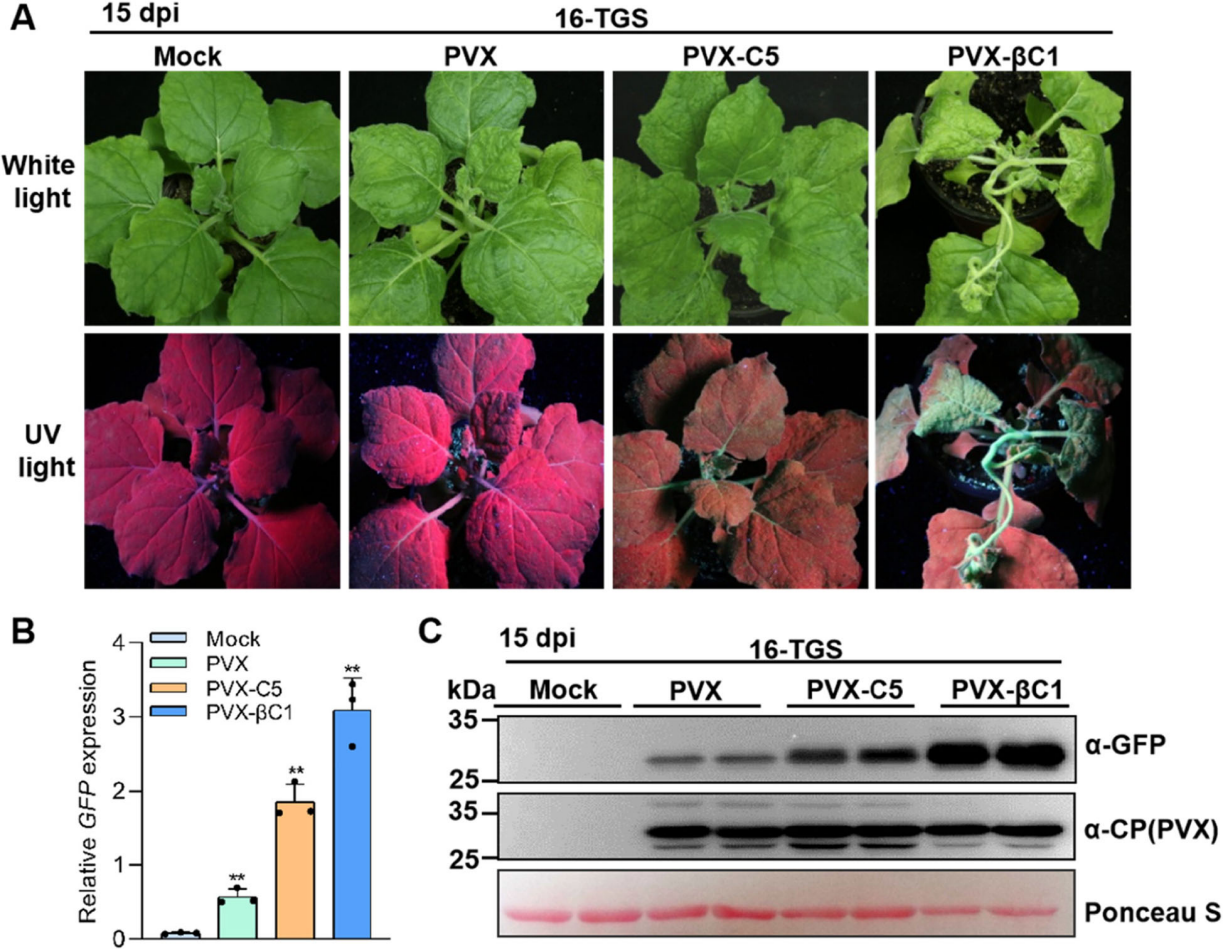
C5 reverses the transcriptional silencing of GFP. **A** Symptoms of 16-TGS *N. benthamiana* plants inoculated with Mock, PVX, PVX-C5, or PVX-βC1 (as positive control) under white light or UV light at 15 dpi. **B** RT-qPCR analysis of relative *GFP* expression levels in the agroinfiltrated leaf patches from (**A**). Three plants were analyzed for each treatment and this experiment was repeated three times with similar results. Error bars represent ± SD (*n* = 3), and *NbActin2* was used as internal reference. Student’s *t* test was used to statistically analyze each group of data, and double asterisks indicate significant statistical differences (**p < 0.01) between two treatments. **C** Western blot assays showed the accumulation of GFP and PVX CP in the agroinfiltrated leaf patches as indicated from (**A**). Ponceau S staining of the large RuBisCO subunit serves as loading control

Geminiviruses are susceptible to epigenetic modification, leading to TGS. In order to counteract plant defenses, some geminiviruses encode suppressors of TGS (Zrachya et al., 2007; Luna et al., 2012; Li et al., 2018; Prasad et al., 2020). To clarify whether C5 has the ability to suppress TGS, we inoculated *A. tumefaciens* cultures harboring PVX-C5 into 16-TGS *N. benthamiana* plants, which contain a transcriptionally silenced GFP transgene driven by the CaMV 35S promoter. 16-TGS plants inoculated with *A. tumefaciens* cultures carrying the empty binary vector (Mock) or PVX were used as negative controls, and PVX-βC1-inoculated 16-TGS plants were used as positive controls, respectively. At 15 dpi, we found that the negative controls could not reverse GFP silencing in infected plants, which appeared red under UV light (Fig. 5A). However, the 16-TGS *N. benthamiana* plants inoculated with PVX-C5 and PVX-βC1, showed visible green fluorescence in the upper leaves (Fig. 5A). RT-qPCR and western blot analyses showed that PVX-C5 and PVX-βC1 infected plants accumulated higher levels of GFP RNA and protein (Fig. 5B and C), indicating that C5 is a potential TGS suppressor.

### C5 is important for TYLCV infection in *N. benthamiana* and *Solanum lycopersicum*

**Fig. 6:**
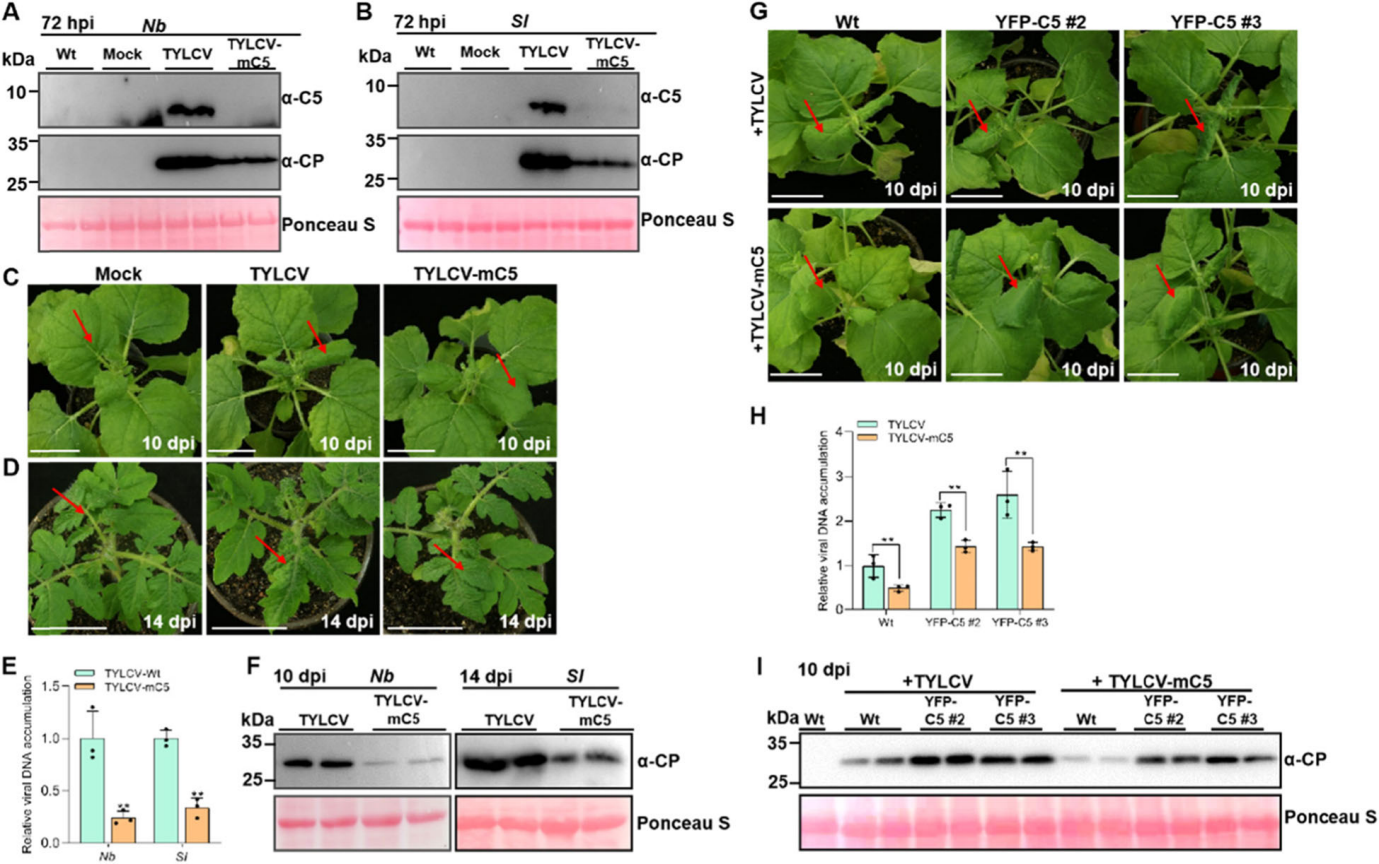
C5 is important for TYLCV infection in *N. benthamiana* and *S. lycopersicum* plants. **A-B** Western blot analyses of TYLCV C5 and CP protein accumulation with specific anti-C5 and anti-CP antibodies in local leaves of TYLCV- and TYLCV-mC5-inoculated plants of *N. benthamiana* (*Nb*) (**A**) or in cotyledons of TYLCV- and TYLCV-mC5- infected plants of *S. lycopersicum* (*Sl*) (**B**). Wt: uninoculated plants; Mock: inoculated plants with pCambia 2300 vector; TYLCV/TYLCV-mC5: inoculated plants with the infectious clones of TYLCV/TYLCV-mC5. Ponceau S staining of the large RuBisCO subunit serves as loading control. **C** Symptoms of *N. benthamiana* plants inoculated with Mock, TYLCV, or TYLCV-mC5 infectious clones at 10 dpi. Bar = 4 cm. **D** Symptoms of tomato plants inoculated with Mock, TYLCV, or TYLCV-mC5 infectious clones at 14 dpi. **E** Western blot analyses of TYLCV CP accumulation in systemic leaves of TYLCV- and TYLCV-mC5- infected plants from (**C** and **D**). Ponceau S staining of the large RuBisCO subunit serves as loading control. **F** qPCR analysis of the viral DNA accumulation of TYLCV or TYLCV-mC5 -infected plants from (**C** and **D**). Three plants were analyzed for each treatment and this experiment was repeated three times with similar results. Error bars represent ± SD (*n* = 3) and 25S RNA was used as the internal reference. **G** Symptoms of YFP-C5 transgenic *N. benthamiana* plants infected with TYLCV or TYLCV-mC5 at 10 dpi. Bar = 4 cm. **H** Western blot analyses of TYLCV CP accumulation in systemic leaves of TYLCV- and TYLCV-mC5-infected plants from (**G**). Ponceau S staining of the large RuBisCO subunit serves as loading control. **I** qPCR analysis of the viral DNA accumulation of TYLCV or TYLCV-mC5 -infected plants from (**G**). Three plants were analyzed for each treatment and this experiment was repeated three times with similar results. Error bars represent ± SD (*n* = 3). Student’s *t* test was used to statistically analyze each group of data, and double asterisks indicate significant statistical differences (**p < 0.01) between two treatments

To investigate whether C5 plays a role in the TYLCV infection of *N. benthamiana* and *S. lycopersicum* plants, we constructed an infectious clone of TYLCV mutated in C5 (TYLCV-mC5), which renders it unable to produce C5. A T862C substitution was introduced into the start codon of C5 by site-directed mutagenesis to block the translation of C5. Of note, the T862C substitution did not change the amino acid sequence of the V1 ORF (CP). The infectious clones of wild-type TYLCV and the mutant TYLCV-mC5 were inoculated into *N. benthamiana* and *S. lycopersicum* plants. At 72 hpi, we used anti-C5 antibodies to detect the presence of the TYLCV C5 protein through western blot analyses. As shown in Fig. 6A-B, the C5 protein was only detected in TYLCV-inoculated, but not in TYLCV-mC5-inoculated *N. benthamiana* and *S. lycopersicum* plants. Of note, the TYLCV CP protein could be detected both in TYLCV- and TYLCV-mC5-inoculated plants, but plants inoculated with the TYLCV-mC5 infectious clones displayed lower accumulation of the CP (Fig. 6A-B). At 10 dpi, the infection of TYLCV-mC5 compared to TYLCV caused milder disease symptoms including milder leaf curling, which was also accompanied by lower accumulation of viral load and CP, as determined by qPCR and western blot analyses (Fig. 6C-F). These results indicate that C5 is an important pathogenicity factor for TYLCV in both *N. benthamiana* and *S. lycopersicum* plants.

To further confirm the virulence function of C5, we obtained transgenic *N. benthamiana* plants expressing YFP-C5 under a 35S promoter. YFP-C5 transgenic *N. benthamiana* plants accumulated high levels of YFP-C5 RNA and protein (Fig. S4A-C), and did not display obvious developmental defects (Fig. S3A). To determine whether YFP-C5 transgenic plants can restore the virulence of TYLCV-mC5, we conducted a complementation assay experiment. The infectious clones of TYLCV and TYLCV-mC5 were inoculated into wild-type and YFP-C5 transgenic *N. benthamiana* plants for monitoring viral symptoms and analyzing the accumulation of viral DNA and protein. As shown in Fig. 6G-I, YFP-C5 transgenic plants inoculated with TYLCV exhibited more severe leaf curling symptoms and accumulated higher levels of viral DNA and protein, indicating that the expression of C5 facilitates viral infection. Furthermore, YFP-C5 transgenic plants inoculated with TYLCV showed higher levels of viral DNA and protein than TYLCV-mC5-infected YFP-C5 transgenic plants (Fig. 6H-I), indicating that overexpression of the C5 protein benefits the accumulation of viral DNA and protein. These data demonstrate that ectopic expression of C5 can enhance the accumulation of TYLCV DNA and protein, and partially restores the virulence of TYLCV-mC5, further supporting that C5 is an important pathogenicity factor of TYLCV.

## Discussion

Our recent report has revealed that TYLCV encodes more than the six canonical proteins, and at least some additional small proteins from TYLCV display specific subcellular localization patterns (Gong et al., 2021). TYLCV C5 (ORF1 in Gong et al., 2021) was found to localize in the nucleus and cytoplasm, but its detailed role in the context of TYLCV infection was not investigated. In this study, the expression and function of TYLCV C5 is extensively studied.

Accumulated evidence shows many geminiviruses, including MYMIV, tomato leaf deformation virus (ToLDeV), ToCMoV, and WmCSV, contain the C5/AC5 ORF in the complementary strand of their genomes (Kheyr-Pour et al., 2000; Fontenelle et al., 2007; Li et al., 2015; Li et al., 2021). Here we also analyzed the conservation of C5/AC5 in different geminiviruses, and revealed that the C5/AC5 ORF is present in many geminiviruses (Fig. S1A), indicating that the C5/AC5 ORF is ubiquitous in this family. The expression of the C5 ORF and accumulation of the C5 protein in the context of TYLCV infection was further confirmed through RT-PCR using C5-specific primers and western blot using an anti-C5 antibody (Fig. 1 and S1). These results provide convincing evidence that the TYLCV C5 ORF encodes a protein rather than being a pseudogene. In addition, the promoter activity of the 500 nt upstream sequences of C5 in the TYLCV genome was also examined in this study (Fig. 2). Although the promoter activity of the C5 upstream sequences is lower than that of the 35S promoter, it activates the expression of exogenous gene GUS or GFP effectively.

TGS and PTGS are two major layers of plant defense responses against geminivirus infection (Raja et al., 2010; Hanley-Bowdoin et al., 2013; Li & Wang, 2019; Prasad et al., 2020; Gui et al., 2022; Guo et al., 2022). It has been reported that geminiviruses encode several suppressors of TGS and PTGS, including C2, V2, C4, and βC1 (Li et al., 2018; Wang et al., 2018; Fondong, 2019; Gnanasekaran et al., 2019; Guerrero et al., 2020). The βC1 protein encoded by betasatellite, which is associated with many monopartite geminiviruses, functions as a pathogenicity factor required for inducing severe symptoms, and as a suppressor of TGS and PTGS (Li et al., 2018; Gnanasekaran et al., 2019). TYLCV is a monopartite geminivirus, which is not associated with betasatellite in natural infections in the field. Therefore, the evolved C5 protein might display a similar role to that of βC1. In this work, our data showed that C5, like βC1, can suppress TGS and PTGS. Interestingly, C5 can only suppress ss-GFP-induced RNA silencing, but fails to suppress dsGFP-induced RNA silencing (Fig. 4), indicating that C5 might suppress RNA silencing by inhibiting the conversion from ssRNA to dsRNA. βC1 has been reported to suppress the generation of primary siRNAs but not secondary siRNAs to suppress ssGFP-induced RNA silencing, which is achieved through reducing transcription of *NbRDR6* (Li et al., 2014) and promoting the degradation of NbSGS3 (Li et al., 2017). Therefore, it is possible that TYLCV C5 might inhibit ssGFP-induced RNA silencing by interfering with the function of RDR6/SGS3 pathway. Here we also showed that a PVX-based vector expressing C5 can induce severe mosaic symptoms and elevated ROS during infection, strongly suggesting that C5 has a role in viral pathogenicity and induction of host defense responses. Strikingly, the lack of C5 negatively affected viral infection in *N. benthamiana* and *S. lycopersicum* plant. However, C5 is not essential, since a C5 null mutant virus can still accumulate and establish a systemic infection. Owing to the limited coding capacity of geminiviruses, their proteins often play multiple roles in the viral life cycle. In this study, we demonstrated that TYLCV encodes a C5 protein, which is a virulence factor and enhances the pathogenicity of PVX in *N. benthamiana*, and is important for the TYLCV infection. In addition, C5 is also a suppressor of TGS and PTGS. Our work expands the repertoire of TYLCV proteins, and sheds light on the roles of C5 from TYLCV in viral infection.

## Materials and methods

### Plant materials

*N. benthamiana* and *S. lycopersicum* plants were grown in a growth chamber set at 60% relative humidity, 16 h: 8 h (light: dark) photoperiod, and 25 °C:18 °C regime. Transgenic GFP 16c seeds were a gift of David C. Baulcombe, and 16-TGS plants were described previously (Buchmann et al., 2009).

### Sequence analysis

C5 ORFs were predicted and identified using the Open Reading Frame Finder software at the NCBI (https://www.ncbi.nlm.nih.gov/orffinder/). Multiple aligned C5 amino acid sequences using the neighbor-joining method 1000 bootstrap replications in MEGA7 and generated a phylogenetic tree for these C5 ORFs. The exact name and accession number of the selected C5 ORFs can be found in a previous report (Li et al., 2015) with the exception of C5 from TYLCV (Accession No: QIH13015).

### Plasmids and constructs

The *C5* gene sequence and promoter sequence were obtained from the TYLCV isolate (MN432609). The 500-nt sequence upstream of the C5 ATG was subcloned into pINT121-GUS using *Hind* III and *Bam*H I sites to produce pINT121-C5-GUS (pC5-GUS), and the sequence was subcloned into pCHF3-GFP at *Eco*R I and *Sac* I sites to produce pCHF3-C5-GFP (pC5-GFP) through In-Fusion Cloning, respectively. The full-length *C5* gene sequence was recombined into pEarleygate 203 vector through Gateway technology, which was used to express Myc-fused C5 protein. The recombinants were then transformed into *A. tumefaciens* EHA105 for agroinfiltration. The full-length *C5* gene sequence was inserted into a PVX-based vector using *Cla* I and *Sal* I sites to produce PVX-C5, and the recombinant was transformed into *A. tumefaciens* GV3101. All primers used in this study was shown in Supplementary Table S1.

### Viral inoculation

The TYLCV isolate used in this study is TYLCV-BJ (MN432609). *A. tumefaciens* cultures carrying infectious clones of TYLCV and TYLCV-mC5 were adjusted to optical density (OD_600_) of 0.6 in infiltration buffer (10 mM MES, 10 mM MgCl_2_, and 100 μM acetosyringone) before being infiltrated into *N. benthamiana* leaves at the six-leaf stage or *Solanum lycopersicum* true-leaf at the two-leaf stage. For inoculation of PVX and PVX-based recombinants, the agrobacterium cultures carrying PVX, PVX-C5 or PVX-βC1 were resuspended in infiltration buffer to an OD_600_ = 1.0 before infiltration into wild-type *N. benthamiana*, 16-TGS or 16c plants. For PTGS and TGS suppression assays, details can be found in previous reports (Xiong et al., 2007; Yang et al., 2011; Li et al., 2015).

### DNA extraction and qPCR

Genomic DNA was extracted from collected plant leaves through the CTAB method (Akbergenov et al., 2006). Viral DNA accumulation was measured by qPCR assays with TB Green Premix Ex Taq II (Takara, Japan). 100 ng gDNA was used in each 20 μL reaction. 25S RNA was selected as an internal reference for the assays.

### RNA extraction and RT-qPCR

Total RNA was extracted from collected plant leaves using TRIzol reagent. The total RNA was reverse transcribed to complementary DNA (cDNA) using the PrimeScript RT reagent Kit (Takara, Japan). Relative gene expression levels were measured by RT-qPCR assays with specific primer pairs (Table S1) on QuantStudio Real- Time PCR System (ABI Q6). *NbActin2* gene was selected as an internal reference for the assays.

### 5′ rapid amplification of cDNA ends (RACE)

Total RNA was extracted from TYLCV-infected *N. benthamiana* leaves with TaKaRa MiniBEST Universal RNA Extraction Kit (Takara, Japan). High purity RNA was used for cDNA synthesis with SMARTer RACE 5′/3′ Kit (Takara, Japan), and then the RACE products were amplification with nested PCR. The amplified product was ligated into pEASY®-Blunt Simple Cloning T vector (TransGen Biotech, China) for sequencing.

### Confocal microscopy

GFP fluorescence was observed and photographed using laser scan confocal microscopy (LSM980 Carl Zeiss, Germany). GFP was excited at 488 nm, and the emitted light was captured at 500–550 nm. The relative intensity of GFP was measured with the ZEN3.1 software with the method of mean intensity in the same area.

### 3,3′-diaminobenzidine (DAB) staining

The production of H_2_O_2_ in plants was detected through DAB staining. *N. benthamiana* leaves of PVX-infected plants were incubated in DAB solution (1 mg/mL, PH3.8) for 10 h at 25 °C in the dark, then boiled for 5 ~ 10 min and washed by 95% ethanol.

### Protein extraction and western blotting

Total protein was extracted from collected plant leaves using protein extraction buffer (50 mM Tris-HCl (pH 6.8), 9 M carbamide, 4.5% (m/v) SDS, 7.5% (v/v) 2-Mercaptoethanol). Total proteins were separated by 12% sodium dodecyl sulphate­polyacrylamide gel electrophoresis (SDS-PAGE) electrophoresis and transferred to nitrocellulose (NC) membrane. The sample proteins were detected by immunoblotting with primary mouse polyclonal antibodies, followed by anti-mouse IgG HRP secondary antibody. Primary antibodies used in this study are as follows: anti-GFP (1:5000; ROCHE, USA), anti-PVX CP (1:5000) and anti-TYLCV CP (1:5000) (Wu & Zhou, 2005; Wu et al., 2012).

### GUS staining and quantitative GUS activity assay

Infiltrated leaves from GUS-, pC5-GUS- and p35S-GUS- inoculated plants were stained using a GUS stain kit (Coolaber, SL7160). Images were photographed through a stereomicroscope (Olympus SZX16). Quantitative GUS activity assays were performed following the method of Jefferson et al (Jefferson et al., 1987).

## Supplementary Information

Additional file 1: **Supplementary Table 1**. **Supplementary Fig. 1.** Sequence and expression analyses of the TYLCV C5 ORF. **A** Distribution of the C5 ORF in the phylogenic tree based on full genomic sequences of 58 begomoviruses. The exact name and accession number of the selected begomoviruses can be found in a previous report (Mohammad A et al., 2007) except TYLCV-BJ (Accession No. MN432609). **B** Real-time fluorescent quantitative PCR used for absolute quantitative analysis of *V1*, *V2*, *C4* and *C5* gene expression levels. Total RNA was extracted from the TYLCV- inoculated *N. benthamiana* leaves at 60 h post inoculation (hpi) and the systemic leaves of TYLCV infected *N. benthamiana* leaves at 10 days post inoculation (dpi) using V1, V2, C4, and C5-specific primers (Table S1). **C** C5-His fusion protein detected by the purified anti-C5 antibodies. Coomassie brilliant blue-staining of the gel is used as loading control. **Supplementary Fig. 2.** C5 suppresses ssGFP-induced RNA silencing but not dsGFP-induced RNA silencing in 16c *N. benthamiana* plants. **A** Co-infiltrated with *A. tumefaciens* cultures expressing GFP (35S-GFP) and Mock, C5, or P19 in the same leaf of 16c *N. benthamiana* plants, which were photographed under UV light at 4 dpi. **B** Co-infiltrated with double-stranded GFP (35S-dsGFP) and Mock, C5, or P19 in the same leaf of 16c *N. benthamiana* plants, which were photographed under UV light at 4 dpi. **Supplementary Fig. 3.** PVX-expressing C5 suppresses ssGFP induced RNA silencing. **A** The 16c *N. benthamiana* plants co-infiltrated with *A. tumefaciens* cultures expressing GFP (35S-GFP) and PVX, or PVX-βC1, or PVX-C5, were photographed under UV light at 7 dpi and 20 dpi. **B** RT-qPCR analysis of relative *GFP* expression levels in the agroinfiltrated leaf patches from (**A**). Error bars represent ± SD (*n* = 3) and *NbActin2* was used as internal reference. Student’s *t* test was used to statistically analyze each group of data, and double asterisks indicate significant statistical differences (**p < 0.01) between two treatments. **C** Western blot analysis of the accumulation of GFP and PVX CP in the agroinfiltrated leaf patches as indicated from (**A**). Ponceau S staining of the large RuBisCO subunit serves as loading control. **Supplementary Fig. 4.** Characterization of transgenic *N. benthamiana* lines expressing C5. **A** Phenotype of 5-week-old 35S: YFP-C5 T1 transgenic *N. benthamiana* lines (YFP-C5 #2 and YFP-C5 #3) compared with wild type (Wt) *N. benthamiana* plants. Bar = 4 cm. **B** RT-qPCR analysis of relative *C5* expression levels in Wt and YFP-C5 transgenic lines. Error bars represent ± SD (*n* = 3) and *NbActin2* was used as internal reference. Student’s *t* test was used to statistically analyze each group of data, and double asterisks indicate significant statistical differences (**p < 0.01) between two treatments. **C** Western blot showing YFP protein accumulation in the plants from (**B**); Ponceau S staining of the large RuBisCO subunit serves as loading control.

## Data Availability

The authors declare that all data generated or analyzed during this study are included in the article and its supplementary information files.

## Abbreviations

*A. tumefaciens*: *Agrobacterium tumefaciens*
CaMV: Cauliflower mosaic virus
CP: Coat protein
DAB: Diaminobenzidine
dsGFP: Double-stranded GFP
MYMIV: Mungbean yellow mosaic India virus
*N. benthamiana*: *Nicotiana benthamiana*
ORF: Open reading frame
PCD: Programmed cell death
PTGS: Post-transcriptional gene silencing
PVX: Potato virus X
RACE: Rapid amplification of cDNA ends
ROS: Reactive oxygen species
RSV: Rice stripe virus
*S. lycopersicum*: *Solanum lycopersicum*
ssGFP: Single-stranded GFP
TGS: Transcriptional gene silencing
TSS: Transcription start site
ToCMoV: Tomato chlorotic mottle virus
ToLDeV: Tomato leaf deformation virus
TYLCCNB: Tomato yellow leaf curl China betasatellite
TYLCCNV: Tomato yellow leaf curl China virus
TYLCV: Tomato yellow leaf curl virus
WmCSV: Watermelon chlorotic stunt virus
