# Supplementary material for: The novel C5 protein from tomato yellow leaf curl virus is a virulence factor and suppressor of gene silencing
Source: Stress Biol. 2022 Apr 2;2(1):19. doi: 10.1007/s44154-022-00044-3 (PMC10442036; doi:10.1007/s44154-022-00044-3)
Supplement: Supplementary file 1 — Supplementary Table 1. Supplementary Fig. 1. Sequence and expression analyses of the TYLCV C5 ORF. A Distribution of the C5 ORF in the phylogenic tree based on full genomic sequences of 58 begomoviruses. The exact name and accession number of the selected begomoviruses can be found in a previous report (Mohammad A et al., 2007) except TYLCV-BJ (Accession No. MN432609). B Real-time fluorescent quantitative PCR used for absolute quantitative analysis of V1, V2, C4 and C5 gene expression levels. Total RNA was extracted from the TYLCV- inoculated N. benthamiana leaves at 60 h post inoculation (hpi) and the systemic leaves of TYLCV infected N. benthamiana leaves at 10 days post inoculation (dpi) using V1, V2, C4, and C5-specific primers (Table S1). C C5-His fusion protein detected by the purified anti-C5 antibodies. Coomassie brilliant blue-staining of the gel is used as loading control. Supplementary Fig. 2. C5 suppresses ssGFP-induced RNA silencing but not dsGFP-induced RNA silencing in 16c N. benthamiana plants. A Co-infiltrated with A. tumefaciens cultures expressing GFP (35S-GFP) and Mock, C5, or P19 in the same leaf of 16c N. benthamiana plants, which were photographed under UV light at 4 dpi. B Co-infiltrated with double-stranded GFP (35S-dsGFP) and Mock, C5, or P19 in the same leaf of 16c N. benthamiana plants, which were photographed under UV light at 4 dpi. Supplementary Fig. 3. PVX-expressing C5 suppresses ssGFP induced RNA silencing. A The 16c N. benthamiana plants co-infiltrated with A. tumefaciens cultures expressing GFP (35S-GFP) and PVX, or PVX-βC1, or PVX-C5, were photographed under UV light at 7 dpi and 20 dpi. B RT-qPCR analysis of relative GFP expression levels in the agroinfiltrated leaf patches from (A). Error bars represent ± SD (n = 3) and NbActin2 was used as internal reference. Student’s t test was used to statistically analyze each group of data, and double asterisks indicate significant statistical differences (**p < 0.01) between two treatmen [file 44154_2022_44_MOESM1_ESM.docx]

**Supplementary Information**

**Supplementary Table1**

| **Primer name** | **Sequence (5'-3')** |
| --- | --- |
| C5-GSPs-R | GATTACGCCAAGCTTGAAGCAGAATCACACTAATCAGGTC |
| C5-NGSPs-R | GATTACGCCAAGCTTGGTTCGTGATAGAAGGCC |
| pC5-500-Hind III-F | CCCAAGCTTAACCACGACATCATTTCCATT |
| pC5-500-BamH I-R | CGGGATCCGCTACAGTTATTGGTGGGCCC |
| pC5-500-EcoR I-F | CGGAATTCAACCACGACATCATTTCCATT |
| pC5-500-Sac I-R | CGAGCTCGCTACAGTTATTGGTGGGCCC |
| 221-C5-F | GGGGACAAGTTTGTACAAAAAAGCAGGCTTCATGAAATTTCCTCATCACTTGAAAC |
| 221-C5-R | GGGGACCACTTTGTACAAGAAAGCTGGGTCGGTAAAGTCTGGATGGATGAAA |
| IF-C5-Cla I-F | CCATCGATATGAAATTTCCTCATCACTT |
| IF-C5-Sal I-R | ACGCGTCGACGGTAAAGTCTGGATGGATGAA |
| NbActin2-qPCR-F | AAAGACCAGCTCATCCGTGGAGAA |
| NbActin2-qPCR-R | TGTGGTTTCATGAATGCCAGCAGC |
| q-25S-rRNA-F | ATAACCGCATCAGGTCTCCA |
| q-25S-rRNA-R | CCGAAGTTACGGATCCATTT |
| q-TYLCV-F | CCCTCAAAGCTCTATGGCAATCGG |
| q-TYLCV-R | CAGTGACGTCTGTGGAACCCTC |
| q-C5-F | AGGCCTTATGGAAACAGCCC |
| q-C5-R | TCATTCTTCACGGTTGCGGT |
| q-GFP-F | TGAGGGATACGTGCAGGAGA |
| q-GFP-R | TGCCGTTCTTTTGCTTGTCG |

**Supplementary Figures**


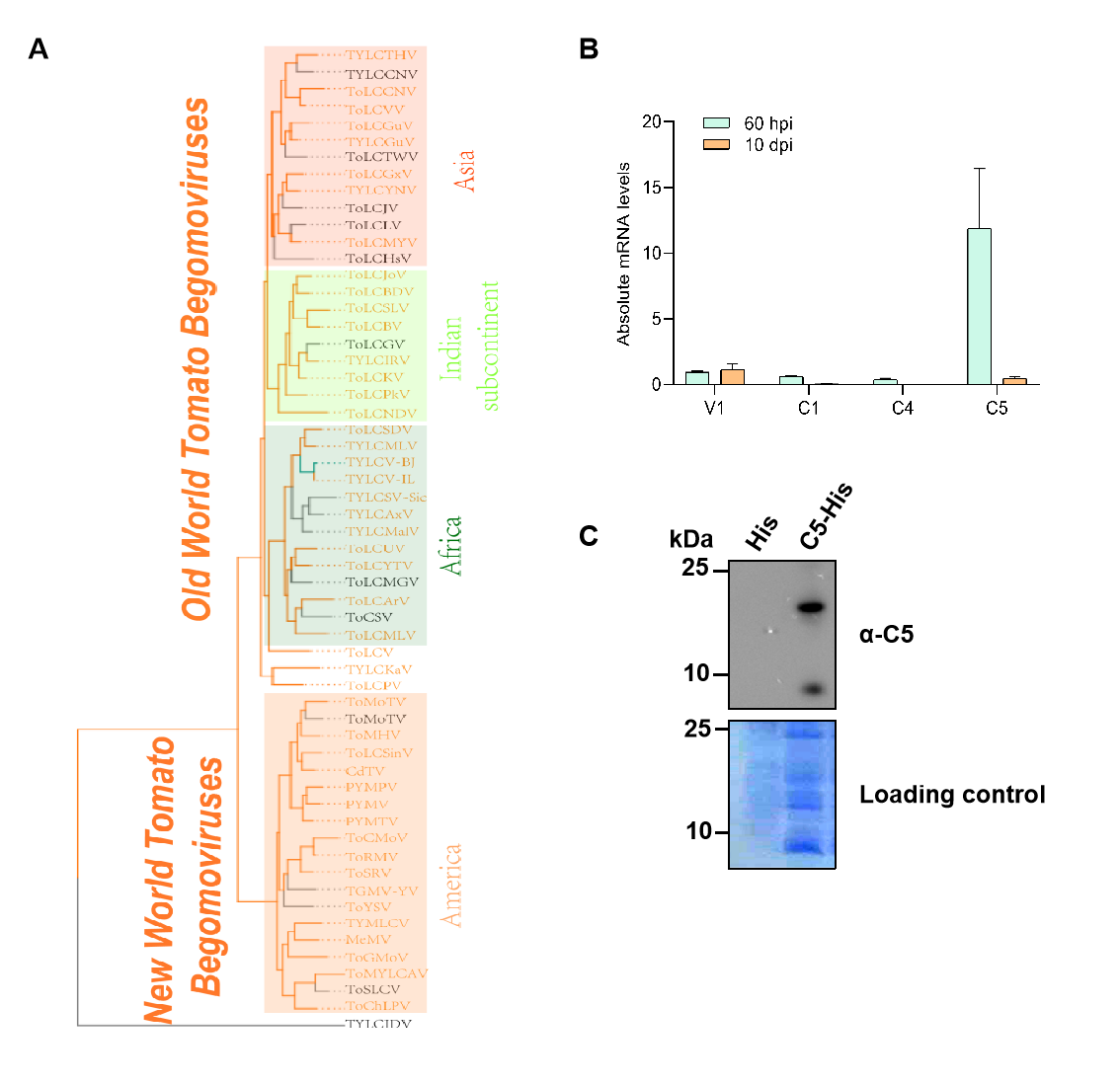


**Supplementary figure 1. Sequence and expression analyses of the TYLCV C5 ORF.** (A) Distribution of the C5 ORF in the phylogenic tree based on full genomic sequences of 58 begomoviruses. The exact name and accession number of the selected begomoviruses can be found in a previous report ([Mohammad A et al., 2007](#_ENREF_32)) except TYLCV-BJ (Accession No. MN432609). (B) Real-time fluorescent quantitative PCR used for absolute quantitative analysis of *V1*, *V2*, *C4* and *C5* gene expression levels. Total RNA was extracted from the TYLCV- inoculated *N. benthamiana* leaves at 60 hours post inoculation (hpi) and the systemic leaves of TYLCV infected *N. benthamiana* leaves at 10 days post inoculation (dpi) using V1, V2, C4, and C5-specific primers (Table S1). (C) C5-His fusion protein detected by the purified anti-C5 antibodies. Coomassie brilliant blue-staining of the gel is used as loading control.


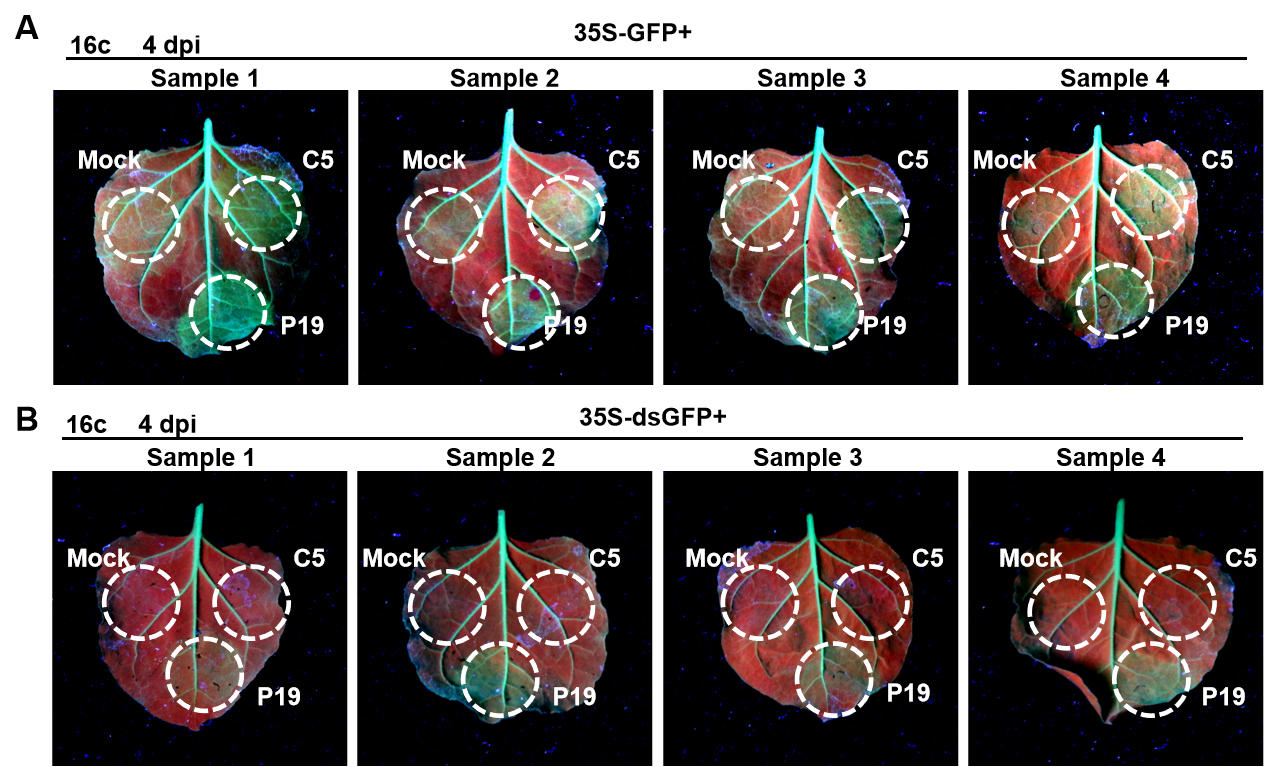


**Supplementary figure 2. C5 suppresses ssGFP-induced RNA silencing but not dsGFP-induced RNA silencing in 16c *N. benthamiana* plants.** (A) Co-infiltrated with *A. tumefaciens* cultures expressing GFP (35S:GFP) and Mock, C5, or P19 in the same leaf of 16c *N. benthamiana* plants, which were photographed under UV light at 4 dpi. (B) Co-infiltrated with double-stranded GFP (35S:dsGFP) and Mock, C5, or P19 in the same leaf of 16c *N. benthamiana* plants, which were photographed under UV light at 4 dpi.


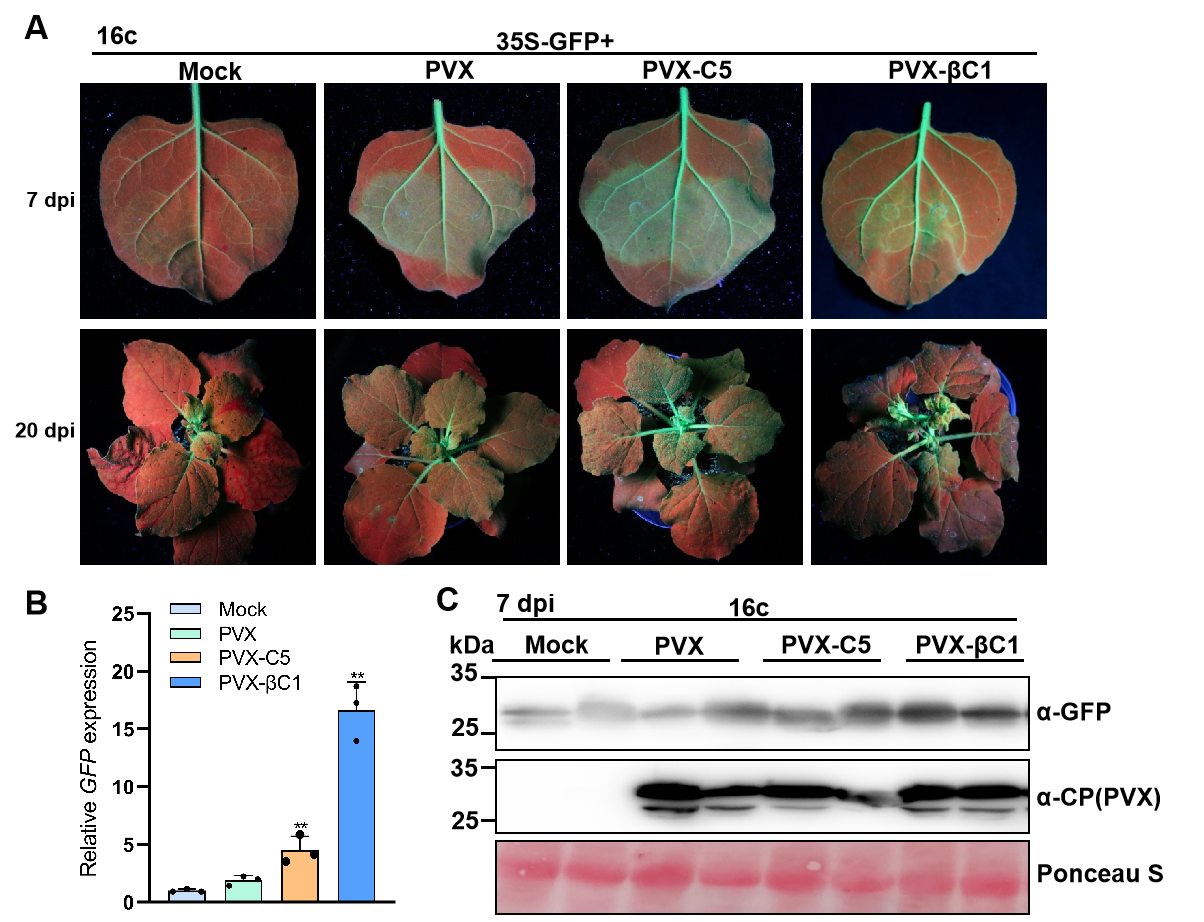


**Supplementary figure 3. PVX-expressing C5 suppresses ssGFP induced RNA silencing.** (A) The 16c *N. benthamiana* plants co-infiltrated with *A. tumefaciens* cultures expressing GFP (35S-GFP) and PVX, or PVX-βC1, or PVX-C5, were photographed under UV light at 7 dpi and 20 dpi. (B) RT-qPCR analysis of relative *GFP* expression levels in the agroinfiltrated leaf patches from (A). Error bars represent ± SD (n=3) and *NbActin2* was used as internal reference. Student’s *t* test was used to statistically analyze each group of data, and double asterisks indicate significant statistical differences (**p<0.01) between two treatments. (C) Western blot analysis of the accumulation of GFP and PVX CP in the agroinfiltrated leaf patches as indicated from (A). Ponceau S staining of the large RuBisCO subunit serves as loading control.


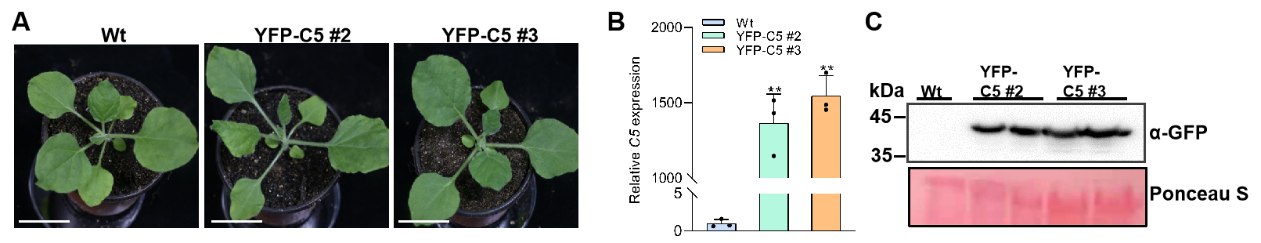
**Supplementary figure 4. Characterization of transgenic *N. benthamiana* lines expressing C5.** (A) Phenotype of 5-week-old 35S: YFP-C5 T1 transgenic *N. benthamiana* lines (YFP-C5 #2 and YFP-C5 #3) compared with wild type (Wt) *N. benthamiana* plants. Bar = 4 cm. (B) RT-qPCR analysis of relative *C5* expression levels in Wt and YFP-C5 transgenic lines. Error bars represent ± SD (n=3) and *NbActin2* was used as internal reference. Student’s *t* test was used to statistically analyze each group of data, and double asterisks indicate significant statistical differences (**p<0.01) between two treatments. (C) Western blot showing YFP protein accumulation in the plants from (B); Ponceau S staining of the large RuBisCO subunit serves as loading control.
